# 

**Published:** 1916-07

**Authors:** 


### CONTENTS.

ORIGINAL COMMUNICATIONS—

The Pulmonic Valve in Pulmonitis.—By Arch Elkin, M. D., Atlanta, Ga. ...... 149

The Chronic Running Ear.—By Ernest S. Colvin, M. D., Atlanta, Ga. .......... 152

EDITORIAL ........................ 162

SELECTIONS AND ABSTRACTS .......... 165

BOOKS REVIEWS ...................... 193

